# The nationwide survey of Japanese public opinion about off-label use of anticancer drugs recommended by comprehensive genomic profiling

**DOI:** 10.1007/s10147-025-02809-y

**Published:** 2025-07-18

**Authors:** Junichi Matsubara, Kumi Mukai, Manabu Muto

**Affiliations:** Department of Clinical Oncology, Kyoto University Hospital, 54 Shogoinkawahara-Cho, Sakyo-Ku, Kyoto, 606-8507 Japan

**Keywords:** Precision medicine, Comprehensive genomic profiling, Molecular targeted therapy, Off-label drug, Nationwide survey

## Abstract

**Background:**

Comprehensive genomic profiling (CGP)-guided precision medicine enables identification of molecular-based recommended therapy (MBRT), including off-label uses of anticancer drugs for rare genomic alterations. However, in Japan, access to such off-label drugs is limited despite their potential therapeutic benefits. This study aimed to investigate public attitudes toward off-label use of anticancer drugs in Japan.

**Methods:**

A nationwide online survey was conducted in Japan from February 15 to 19, 2024, targeting cancer patients (CA), medical professionals (MP), and non-cancer volunteers (non-CA) aged 40 + years. This included explanatory materials on CGP and MBRT, and questionnaires assessing willingness to use off-label drugs in various cost scenarios.

**Results:**

A total of 1,261 responses were analyzed: 419 CA, 430 MP, and 412 non-CA participants (median age: 59, range 40–89). Approximately 80% of MPs reported high comprehension of the explanatory materials (Top-2 box on a 5-point Likert scale), compared with ~ 60% of CA and < 50% of non-CA participants. Willingness to use off-label drugs (Top-2 box) was as follows: “No cost burden”: 51% CA, 62% MP, and 50% non-CA; “Cost ¥200,000 per month (approximately $US1,300)”: 15% CA, 31% MP, and 15% non-CA; “Cost ¥1,000,000 per month (approximately $US6,700)”: 6% CA, 16% MP, and 4% non-CA. Higher comprehension of explanatory materials was associated with greater willingness to use off-label drugs.

**Conclusions:**

Over half of respondents were willing to use off-label anticancer drugs if they were free of charge. However, willingness declined significantly with cost. Policy frameworks are needed in Japan to improve access to CGP-guided off-label therapies.

**Supplementary Information:**

The online version contains supplementary material available at 10.1007/s10147-025-02809-y.

## Introduction

Recent advances in DNA sequencing technology have enabled comprehensive analysis of common and rare genomic alterations in cancer-associated genes [1, 2]. Precision oncology using comprehensive genomic profiling (CGP) by next-generation sequencing has been introduced into clinical practice, expanding to access molecularly targeted therapies for cancer patients [3]. In our prospective study, we evaluated the clinical utility of a CGP testing FoundationOne® CDx (F1CDx, Foundation Medicine, Inc., Cambridge, MA, USA) in patients with previously untreated metastatic or recurrent tumors of gastrointestinal, pancreatic, biliary tract, lung, breast, gynecologic, and melanoma origin (the FIRST-Dx study) [4]. We demonstrated that the molecular tumor board identified molecular-based recommended therapy (MBRT) in 61% of patients, and 29% had actionable genomic alterations listed as companion diagnostics (CDx) in a tumor-agnostic context. Molecularly targeted drugs accompanied by CDx (evidence level A) generally exhibit high efficacy across various tumor types. Evidence level B, C, and D MBRT which are also frequently encountered in both the FIRST-Dx study (33%) [4] and routine clinical CGP practice, are not covered by Japanese National Health Insurance. In contrast, in the United States, the off-label use of approved drugs is commonly determined by the clinical judgment of medical doctors and informed consent of patients. For patients facing life-threatening conditions, the off-label use of approved drugs should be considered in Japan.

To establish a new drug access framework for off-label use of anticancer drugs in Japan, understanding public awareness and demand is crucial. Therefore, we conducted a nationwide survey to assess the Japanese population’s preferences regarding the potential use of off-label anticancer drugs to provide empirical data to inform future policy development.

## Materials and methods

### Study design and participants

A nationwide online survey was conducted in Japan from February 15 to 19, 2024. The survey targets were cancer patients (CA), medical professionals (MP), and non-cancer volunteers (non-CA) aged 40 years and older. Participants were recruited through Mighty Monitor (INTAGE Inc., Tokyo, Japan), an online survey panel operated by a marketing research company. During recruitment, we did not adjust the number of participants based on their residential areas.

The survey included explanatory materials regarding MBRT identified by a CGP test, along with a questionnaire assessing options on the off-label use of anticancer drugs in relation to cost. The aim of this survey was to explore the preferences of a diverse Japanese population regarding the potential off-label use of anticancer drugs.

In our survey, cancer patients were defined as individuals who selected “any solid tumors” as a current disease in the screening question. Solid tumors included gastrointestinal cancer, hepatobiliary and pancreatic cancer, lung cancer, breast cancer, urological cancer (kidney, urinary tract, bladder), gynecological cancer (uterus, ovary), or other solid tumors (oral cavity, pharynx, skin, thyroid gland, brain tumor, etc.). Medical professionals included medical doctors, nurses, pharmacists, and clinical laboratory technicians. Non-cancer volunteers were defined as individuals who did not select either “cancer patients” or “medical professionals” in the screening question. Participants in the survey were required to agree to treat all information and questions obtained through the survey, as well as all related data, as confidential. They also agreed not to disclose this information (including posting on the internet), share it with third parties, or copy or photograph the survey screen.

This study was supported by the Japan Agency for Medical Research and Development (Grant no: JP23lk0221166).

### Explanatory materials on molecular-based recommended therapy and off-label use of anticancer drugs

The explanatory materials on MBRT consisted of four pages (Supplementary Fig. S1), summarized as follows

- Page 1: Options of cancer treatment.
  There are three primary treatment options for cancer: surgery, anticancer drugs (including chemotherapy, molecularly targeted drug, and immune checkpoint inhibitor), and radiation therapy. Treatment selection depends on the type of cancer, the degree of extension of cancer, and whether the case involves an initial diagnosis or recurrence.
- Page 2: Anticancer treatments based on genomic alteration.
  CGP uses cancer tissue obtained through surgery or other procedures to analyze alterations in hundreds of genes in a single test. Recently, the identification of genomic abnormalities in cancer-related genes has made it possible to select anticancer drugs that are more likely to be effective against specific molecular targets.
- Page 3: Japanese healthcare system and off-label drug use.
  Even if the CGP test results identify genomic alterations in a particular cancer, appropriate drugs may not always be accessible under the Japanese insurance system. While some countries allow off-label drug use and have systems such as compassionate use (single patient IND), this is not available in Japan. Off-label drug use refers to the use a drug already approved in Japan beyond the scope of its authorized indications, dosage, or administration described in the package insert.
- Page 4: Consideration for off-label drug use.
  Anticancer drug treatments for unresectable solid tumors are generally intended to slow disease progression of the cancer rather than achieve a cure. (However, in some cases, treatment may suppress cancer progression for a certain period.) Off-label drugs may cause adverse effect similar to those of approved anticancer drugs. As off-label drug use is not covered by Japanese public health insurance, patients are required to bear the full out-of-pocket cost of treatment.

### Questionnaires on the off-label use of anticancer drugs

1. How well did you understand the explanatory materials about MBRT? (Questions for each page.) Responses were rated on a 5-point Likert scale: Excellent, Very well, Fair, Poor, and Very poor. The Top-2 box was defined as the proportion of respondents who selected Excellent or Very well.
2. In diagnosis with cancer, would you consider utilizing an off-label anticancer drug as MBRT identified by CGP testing (depending on the drug cost burden)?
  Responses were rated on a 5-point Likert scale: Very positive, Positive, Fair, Negative, and Not at all. The Top-2 box was defined as the proportion of respondents who selected Very positive and Positive.
3. Please provide the reasons for your answers in Question 2.
  Based on responses to Question 1, overall comprehension of the 4-page explanatory material was classified into three categories: “GOOD”, “FAIR”, and “POOR”. “GOOD” was defined as respondents who answered Excellent or Very well for all pages. “POOR” was defined as those who answered Fair, Poor or Very poor for all pages. “FAIR” included all remaining respondents who did not meet the criteria for “GOOD” or “POOR”.

### Statistical analysis

All data were collected through Mighty Monitor website (INTAGE Inc., Tokyo, Japan). As this survey was exploratory in nature and no predefined hypotheses were established, the target sample size was set at approximately 400 participants per target participant group, based on budgetary constraints. Participants assignment was conducted to ensure an approximately equal distribution of male and female participants across all groups. Difference in response frequencies were analyzed using the standard chi-squared test. A two-sided significance level was set to 0.05. All statistical analyses were performed using the JMP® Pro software (version 17.0.0) (JMP Statistical Discovery LLC, NC, USA).

## Results

From February 15 to 19, 2024, a total of 1,261 participants were recruited for the nationwide survey. Participants characteristics are summarized in Table 1. The median age was 63 years for CA participants, 54 for MP participants, and 59 for non-CA participants. MP exhibited a younger median age which is attributable to the predominance of working-age individuals (≤ 60 years) among participants. There were no notable differences among the participants in terms of marital status or number of children in the household.

**Table 1:** Characteristics of survey participants

	Total	Cancer patients	Medical professionals	Non-cancer volunteers
Number of people	1261	419	430	412
Age, median [range]	59 [40–89]	63 [40–89]	54 [40–77]	59 [40–88]
Gender, N (%)				
Male	654 (52)	213 (51)	236 (55)	205 (50)
Female	607 (48)	206 (49)	194 (45)	207 (50)
Occupation, N (%)				
Medical doctor	220 (17)	10 (2)	210 (49)	0
Nurse	168 (13)	13 (3)	155 (36)	0
Pharmacist	61 (5)	10 (2)	51 (12)	0
Clinical laboratory technician	16 (1)	2 (1)	14 (3)	0
Others	796 (63)	384 (92)	0	412 (100)
Marital status, N (%)				
Married	884 (70)	306 (73)	311 (72)	267 (65)
Unmarried	377 (30)	113 (27)	119 (28)	145 (35)
Number of children in family, N (%)				
0	786 (62)	290 (69)	229 (53)	267 (65)
1	257 (20)	80 (19)	96 (22)	81 (20)
2	146 (12)	38 (9)	61 (14)	47 (11)
3	56 (4)	7 (2)	39 (9)	10 (2)
4 or more	16 (1)	4 (1)	5 (1)	7 (2)
Region in Japan, N (%)				
Hokkaido	63 (5)	18 (4)	30 (7)	15 (4)
Tohoku	96 (8)	35 (8)	34 (8)	27 (7)
Northern Kanto	64 (5)	19 (5)	21 (5)	24 (6)
Tokyo/Kanagawa/Chiba/Saitama	405 (32)	140 (33)	106 (25)	159 (39)
Hokuriku	73 (6)	21 (5)	27 (6)	25 (6)
Tokai	113 (9)	38 (9)	44 (10)	31 (8)
Kinki	215 (17)	75 (18)	83 (19)	57 (14)
Chugoku	76 (6)	26 (6)	19 (4)	31 (8)
Shikoku	38 (3)	11 (3)	13 (3)	14 (3)
Kyushu	118 (9)	36 (9)	53 (12)	29 (7)

The medical history of survey participants is presented in Supplementary Fig. S2A. Among the MP group, approximately half were medical doctors (49%), followed by nurses (36%), pharmacists (12%), and clinical laboratory technicians (3%). Among the CA group, the three most common cancer types were breast cancer (N = 136), gastrointestinal/hepatobiliary/pancreas cancer (N = 127), and urinary cancer (N = 88, Supplementary Fig. S2B). Notably, 35 participants (8%) in the CA group also reported working as medical professionals.

### Comprehension levels of explanatory materials: Responses to “How well did you understand the explanatory materials about MBRT?”

Across the total population, more than 60% of participants demonstrated good comprehension of each explanatory material as assessed by Top-2 box responses on a 5-point Likert scale (Table 2). For “Options of cancer treatment,” 71% of CA participants, 81% of MP participants, and 50% of non-CA participants selected Top-2 box responses (Excellent or Very well). For “Anticancer treatments based on the cancer genomic alteration,” the corresponding proportion in Top-2 box were 57% of CA participants, 80% of MP participants, and 44% of non-CA participants, respectively. For “The Japanese healthcare system and off-label drug use,” 57% of CA participants, 77% of MP participants, and 47% of non-CA participants indicated high levels of understanding. For “Notes on treatment with the off-label drug use,” Top-2 box responses were 60% of CA participants, 78% of MP participants, and 49% of non-CA participants.

**Table 2:** Comprehension levels of explanatory materials

	Total		Cancer patients		Medical professionals		Non-cancer volunteers	
	N	%	N	%	N	%	N	%
Number of people	1261	100	419	100	430	100	412	100
*Options of cancer treatment*								
Excellent	96	8	27	6	57	13	12	3
Very well	761	60	274	65	294	68	193	47
Fair	364	29	107	26	73	17	184	45
Poor	36	3	11	3	4	1	21	5
Very poor	4	0	0	0	2	1	2	1
*Anti-cancer treatments based on the cancer genomic alteration*								
Excellent	83	7	21	5	51	12	11	3
Very well	677	54	216	52	294	68	167	41
Fair	432	34	153	37	79	18	200	49
Poor	63	5	27	6	4	1	32	8
Very poor	6	1	2	1	2	1	2	1
*Japanese healthcare system and the off-label drug use*								
Excellent	84	7	18	4	52	12	14	3
Very well	678	54	220	53	278	65	180	44
Fair	424	34	147	35	91	21	186	45
Poor	70	6	33	8	7	2	30	7
Very poor	5	0	1	0	2	1	2	1
*Notes for treatment with the off-label use of drugs*								
Excellent	83	7	19	5	49	11	15	4
Very well	701	56	229	55	288	67	184	45
Fair	409	32	141	34	86	20	182	44
Poor	64	5	29	7	6	1	29	7
Very poor	4	0	1	0	1	0	2	1

Overall comprehension of the 4-page explanatory material was “GOOD” for 651 participants, “FAIR” for 288 participants, and “POOR” for 322 participants.

### Willingness to utilize off-label drugs: Responses to “Would you consider utilizing the off-label anticancer drug as MBRT identified by CGP testing?”

Participants were asked about their willingness to use off-label anticancer drugs in three different cost conditions: (1) no cost burden, (2) ¥200,000 per month (approximately $US1,300), and (3) ¥1,000,000 per month (approximately $US6,700) drug cost burden. Responses in the Top-2 box on a 5-point Likert scale (Very positive or Positive) were categorized as willing to use.

**Fig. 1:**
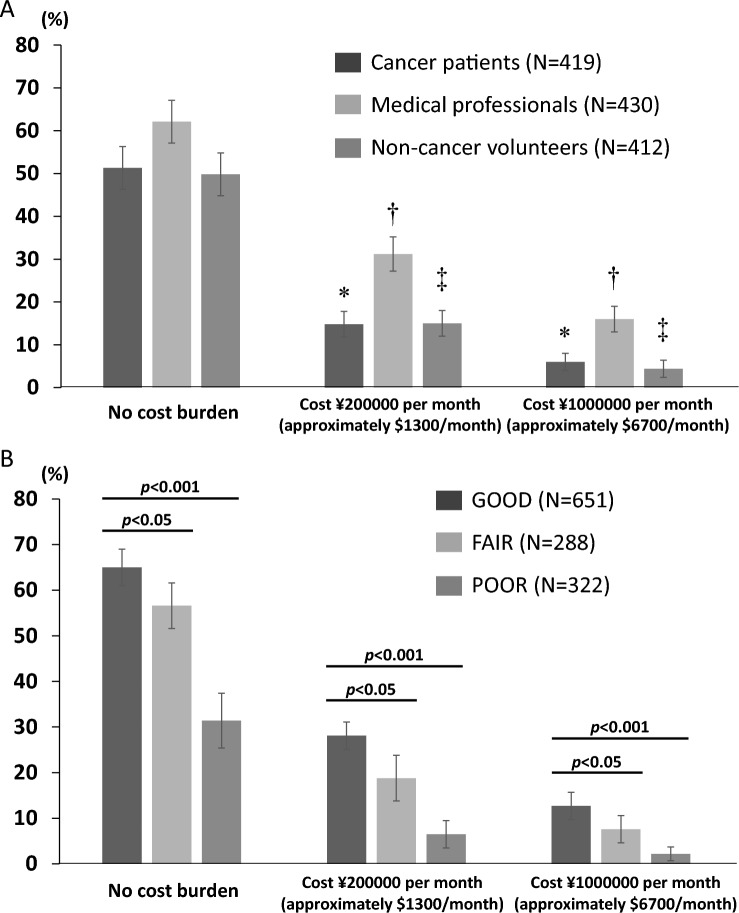
The percentage of participants who would like to use the off-label drug according to cost burden. A, Vertical bar graphs show the percentage of those willing to use the off-label drug—cancer patients (dark gray), medical professionals (light gray), and non-cancer volunteers (gray)—according to the cost burden per month (zero, ¥200,000, and ¥1,000,000). *p < 0.001 vs no cost burden in cancer patients. †*p* < 0.001 vs no cost burden in medical professionals. ‡*p* < 0.001 vs no cost burden in non-cancer volunteers. B, Willingness to utilize off-label drugs according to overall comprehension of explanatory materials. Vertical bar graphs show the percentage of individuals willing to use the off-label drug in GOOD overall comprehension (dark gray), FAIR overall comprehension (light gray), and POOR overall comprehension (gray) according to the cost burden per month (zero, ¥200,000, and ¥1,000,000). Error bars (**A**, **B**) represent the 95% confidence interval.

The results showed that approximately half or more of the participants in all groups expressed willingness to use off-label drugs when there was no cost burden (Fig. 1A and Table 3). This was most prominent among MP participants (62%, 95% confidence interval [CI] 57–67%), compared with 51% (95% CI 46–56%) of CA participants and 50% (95% CI 45–55%) of non-CA participants.

**Table 3:** Willingness to utilize the off-label drug as molecular-based recommended therapy identified by comprehensive genomic profiling

	Total		Cancer patients		Medical professionals		Non-cancer volunteers	
	N	%	N	%	N	%	N	%
Number of people	1261	100	419	100	430	100	412	100
*No cost burden*								
Very positive	157	13	52	12	65	15	40	10
Positive	530	42	163	39	202	47	165	40
Fair	473	38	169	40	136	32	168	41
Negative	85	7	34	8	22	5	29	7
Not at all	16	1	1	0	5	1	10	2
*Cost ¥200,000 per month (approximately $US1,300/month)*								
Very positive	40	3	11	3	20	5	9	2
Positive	218	17	51	12	114	27	53	13
Fair	566	45	198	47	179	42	189	46
Negative	341	27	131	31	94	22	116	28
Not at all	96	8	28	7	23	5	45	11
*Cost ¥1,000,000 per month (approximately $US6,700/month)*								
Very positive	19	2	6	1	10	2	3	1
Positive	93	7	19	5	59	14	15	4
Fair	460	37	137	33	161	37	162	39
Negative	422	34	166	40	123	29	133	32
Not at all	267	21	91	22	77	18	99	24

In contrast, the proportion of participants willing to use the off-label drug declined significantly as the cost burden increased, with similar trends across all participant groups (Fig. 1A and Table 3). When the cost was ¥200,000 per month, 15% (95% CI 12–19%) of CA participants, 31% (27–36%) of MP participants, and 15% (12–19%) of non-CA were willing to use the off-label drug (*p* < 0.001 vs no cost burden in each participant group). When the cost increased to ¥1,000,000 per month, willingness further declined to 6% (4–9%) of CA participants, 16% (13–20%) of MP participants, and 5% (3–7%) of non-CA participants (*p* < 0.001 vs no cost burden in each participant group).

The proportion of participants willing to use the off-label drug with no cost burden was similar between men and women across all groups (Supplementary Table S1). However, when the cost exceeded ¥200,000 per month, the proportion of women who were willing to use the drug decreased more markedly than that of men. A decline in willingness to use the off-label drug with an increasing cost burden was observed across all ages of participants, indicating that this trend was independent of age.

Participants were also asked to provide reasons for their responses to the question on willingness to use off-label anticancer drugs. The most common reason among those who selected Top-2 box responses (Very positive or Positive) was “I expect the anti-tumor efficacy,” cited by 60–75% of respondents (Table 4). This was followed by “I don’t want to do nothing” and “I believe I can contribute to future cancer treatment.” These trends were consistent across all cost burden options. While many MP expressed willingness to pay higher costs in expectation of anti-tumor efficacy, CA and non-CA showed low willingness to do so (Supplementary Table S2). Among participants who responded Fair, Negative, or Not at all, the most frequently cited reason was cost, followed by “uncertain efficacy,” “concerns about safety,” “cancer is incurable,” and “psychological resistance to off-label drug drugs” (Table 4). These trends were also consistent across all cost burden conditions in each participant group (Supplementary Table S2).

**Table 4:** Reasons for answers about willingness to utilize the off-label drug as molecular-based recommended therapy

	No cost burden		Cost ¥200,000 per month (approximately $US1,300/month)		Cost ¥1,000,000 per month (approximately $US6,700/month)	
	N	%	N	%	N	%
*Reasons for* “*very positive” and “positive”*						
Total number pf people	687	100	258	100	112	100
Expect the anti-tumor efficacy	458	67	192	74	67	60
Hate doing nothing	216	31	67	26	28	25
Believe I can contribute to future cancer treatment	193	28	62	24	26	23
Think my family would like it	98	14	37	14	18	16
Others	4	1	4	2	2	2
Don’t know/Don’t want to answer	38	6	13	5	12	11
*Reasons for “fair”, “negative”, and “not at all”*						
Total number pf people	574	100	1003	100	1149	100
Too expensive	NA	NA	594	59	843	73
Safety concerns	183	32	181	18	177	15
Not clear about the efficacy	245	43	301	30	312	27
Cancer can’t be cured	151	26	206	21	214	19
Psychological resistance to using off-label drugs	149	26	181	18	148	13
Think my family would not like it	10	2	23	2	23	2
Others	18	3	15	2	17	2
Don’t know/Don’t want to answer	125	22	123	12	116	10

Abbrebiation *NA* not applicable

### Willingness to utilize off-label drugs according to overall comprehension of explanatory materials

The level of overall comprehension of the explanatory materials demonstrated a significant positive correlation with the proportion of individuals willing to use off-label anticancer drugs (Fig. 1B). In the no-cost burden condition, willingness to use off-label drugs was significantly higher in the GOOD comprehensive participants (65%, 95% CI 61–69%) compared with the “FAIR” participants (57%, 95% CI 51–62%, *p* < 0.05) and the “POOR” participants (31%, 95% CI 23–37%, *p* < 0.001). This trend was consistently observed across both the no-cost burden and higher-cost burden conditions, with a statistically significant increase in willingness to use off-label drugs correlated with higher levels of comprehension of the provided information (Fig. 1B). Similar trends were also observed within each participant group: CA, MP, and non-CA (Supplementary Fig. S3A, B, C).

## Discussion

The nationwide online survey conducted in Japan revealed that approximately half or more of the participants in all three groups (CA, MP, and non-CA) expressed willingness to use off-label anticancer drug if there was no-cost burden (Fig. 1A). In contrast, the proportion of participants willing to use such drugs decreased significantly as the cost burden increased, with a consistent trend observed across all participant groups. To the best of our knowledge, this is the first nationwide survey in Japan to assess public opinion regarding the use of off-label anticancer drugs. The results of this study may serve as a foundation for developing a new drug access system to off-label anticancer therapies in Japan.

The level of comprehension regarding off-label drug use in cancer therapy demonstrated a significant positive correlation with the proportion of individuals willing to use off-label anticancer drugs, as shown in Fig. 1B. Furthermore, the primary reasons for unwillingness to use off-label drugs included concerns about efficacy and safety, psychological resistance to off-label drug use, and financial burden (Table 4). To facilitate the development of a new drug access system for off-label drugs in the future, it will be essential to promote public understanding of molecularly matched cancer treatments across a broad segment of the Japanese population. In addition, governmental initiatives regarding health insurance coverage and regulatory flameworks for off-label drug use may be necessary to reduce patient anxiety and increase access to such treatments.

The survey has several limitations that should be acknowledged. First, in this survey, information about CGP and off-label drug use was explained in a self-study format, but in normal medical practice, explanations are given in person, so potentially, the level of understanding was lower in the CA and non-CA groups than in the MP group. If careful explanations were given in person, the level of understanding could be improved. Furthermore, if the comprehension level improves, the proportion of participants willing to use off-label anticancer drugs may also increase. Second, the present survey did not collect data on cancer stage, presence of active disease in participants in the CA group, or direct involvement of participants in the MP group in cancer genomic medicine. The detailed characteristics of the CA and MP groups could potentially be linked to the responses in this survey. Third, participants aged 40 years and older were recruited based on the assumption that younger individuals might have difficulty perceiving cancer as a personal health concern. As a result, the median age in CA participants was 63 years, which was higher than that of the other participant groups. It should be noted that older individuals are generally more likely to avoid taking risks in medical treatment and to be more sensitive to financial burdens. Similarly, restricting participation to those aged 40 and older might risk under-representing young adults who may face greater economic constraints. We believe that it would be challenging to generalize healthcare perceptions across generations because of varying experiences and economic backgrounds. Fourth, the number of participants in each group was approximately 400. A larger-scale survey may be needed to more accurately capture the true consensus of public opinion in Japan.

In conclusion, the result of the nationwide survey indicated that willingness to use off-label anticancer drugs increased significantly when there was no cost burden, and that comprehension of off-label drug use was positively corelated with willingness to use such treatments. Currently in Japan, patients must bear 100% of medical costs when using off-label drugs outside clinical trials. As CGP testing becomes more widespread in Japan, the chances of off-label drug recommendation of off-label use based on CGP results are expected to increase [4, 5]. To ensure access to effective therapies as “precision medicine,” including those for off-label use, there is a growing need to establish systems such as expanded access programs for individual patients (e.g., single patient IND) or a compassionate use program provided by pharmaceutical companies in Japan.

## Supplementary Information

Below is the link to the electronic supplementary material.

Supplementary file1 (PDF 983 KB) Supplementary Fig S1. Explanatory materials about molecular-based recommended therapy and off-label use of anticancer drugs. The materials were written in Japanese.

Supplementary file2 (PDF 77 KB) Supplementary Fig S2. Medical backgrounds and diseases of participants. A, The medical history of survey participants. Horizontal bars indicate the number of participants in groups of cancer patients (yellow), medical professionals (blue), and non-cancer volunteers (green). B, The distribution of cancer types in cancer patients. The numbers in the pie chart denote the number of patients.

Supplementary file3 (PDF 79 KB) Supplementary Fig S3. Willingness to utilize off-label drugs according to overall comprehension of explanatory materials and cost burden. Vertical bar graphs show the percentage of individuals willing to use the off-label drug in GOOD overall comprehension (dark gray), FAIR overall comprehension (light gray), and POOR overall comprehension (gray) according to the cost burden per month (zero, \200,000, and \1,000,000) in cancer patient participants (A), medical professional participants (B), and non-cancer volunteer participants (C).

Supplementary file4 (XLSX 26 KB) Supplementary Table S1. Willingness to utilize the off-label drug as molecular-based recommended therapy identified by comprehensive genomic profiling according to gender and age. Supplementary Table S2. Reasons for answers about willingness to utilize the off-label drug (according to participants’ backgrounds).

## Data Availability

Data are not publicly available in order to protect participant privacy.

## Abbreviations

CDx: Companion diagnostics
CGP: Comprehensive genomic profiling
CI: Confidence interval
F1CDx: FoundationOne® CDx
MBRT: Molecular-based recommended therapy
