# Supplementary material for: The nationwide survey of Japanese public opinion about off-label use of anticancer drugs recommended by comprehensive genomic profiling
Source: Int J Clin Oncol. 2025 Jul 18;30(9):1692–9. doi: 10.1007/s10147-025-02809-y (PMC12378634; doi:10.1007/s10147-025-02809-y)
Supplement: Supplementary file 1 — Supplementary file1 (PDF 983 KB) Supplementary Fig S1. Explanatory materials about molecular-based recommended therapy and off-label use of anticancer drugs. The materials were written in Japanese. [file 10147_2025_2809_MOESM1_ESM.pdf]

Supplementary Fig. S1

がんの治療法について

Options of cancer treatment

■がんの代表的な治療法は、手術治療、薬物療法（抗がん剤治療）、がん免疫療法、放射線治療の４つがあります。

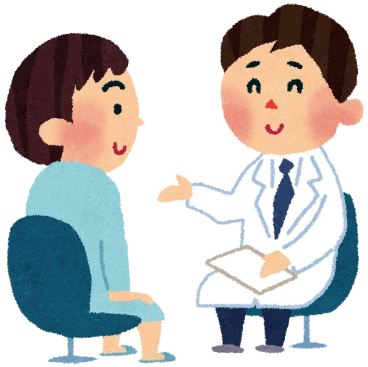

■がんの治療は、がんの種類や進行度、初めての治療か2回目以降の治療かなどを考慮して、治療法が選択されます。

■治療法を選択するにあたっての一般的な考え方は次のようになります。

- **手術治療：**がんを切り取ることができる場合は、手術により治癒を目指します。
- **薬物療法（抗がん剤治療）：**がんが全身に広がっていれば、がん薬物療法でがんの進行を遅らせることを目指します。手術前後に実施して、治療成績を向上させる場合もあります。
- **放射線治療：**手術が難しい場合、放射線治療が選択されることもあります。

※ 上記はあくまでも一般的な考え方であり、患者さん個々の状況により治療選択は異なります。

Supplementary Fig. S1

遺伝子の異常に応じた抗がん剤治療について

Anticancer treatments based on the cancer genomic alteration

■がんの原因は、遺伝子の異常、すなわち遺伝子に傷が入ることだとされています。

■**がん遺伝子パネル検査**は、手術などで採取されたがんの組織を用いて、1回の検査で多数（多くは100以上）の遺伝子について、異常があるかどうかを調べることができます。

■最近では、がんに関連する遺伝子の異常の種類を調べることで、**その遺伝子異常に効きやすい抗がん剤を用いることができるようになってきています。**

遺伝子の異常 A
がある患者さん
→
遺伝子異常Aに効きやすい
薬剤 A

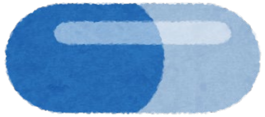

遺伝子の異常 B
がある患者さん
→
遺伝子異常Bに効きやすい
薬剤 B

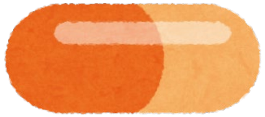

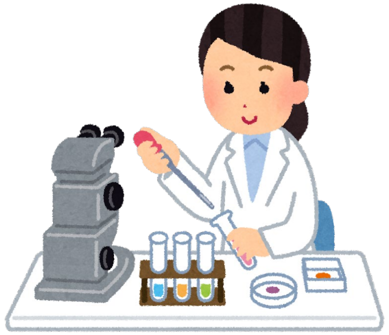

Supplementary Fig. S1

Japanese Healthcare System and the off-label drug use

保険適用薬がない遺伝子の異常が見つかった場合について

■がん遺伝子パネル検査の結果、自分におきているがんの遺伝子異常の種類が分かったとしても、日本の保険制度上、保険適用となる薬がない場合もあり、自分に合う薬を使用できるとは限りません。

■そのような場合、海外と日本では制度上の違いがあります。

- **海外（一部の国）：**見つかった遺伝子の異常に効くかもしれない薬があれば、**適応外治療薬の使用***や人道的な治験**への参加を検討できる場合があります。
- **日本：**基本的にそのような制度はありません。

***適応外治療薬の使用とは、すでに国内で承認されている医薬品を、承認内容の範囲外、すなわち添付文書に記載されている効能・効果、用法・用量の範囲外で使用することです。**

**治験とは、まだ国に承認されていない薬を用いて、薬の有効性や安全性に関するデータを収集するために行われる人を対象とした試験です。

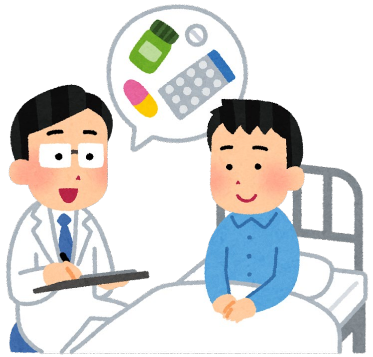

Supplementary Fig. S1

適応外治療薬の使用に関する注意について

Notes for treatment with the off-label use of drugs

■ がん薬物療法は、がんの進行を遅らせることを目的としており、**適応外治療薬を使用する場合でも基本的にがんが治るわけではありません。（ただし、治療により一定期間がんの進行をくい止めることが期待できます。）**

■ 適応外治療薬を使用する場合でも、**通常のがん薬物療法と同様に、副作用が発現する可能性があります。**

■ 適応外治療薬を使用する場合、公的保険の対象にならないため、**治療にかかる費用を全額自己負担する必要があります。**ただし、自由診療保険など民間保険に加入している場合は、民間保険から支払われる場合もあります。
