# Supplementary material for: The nationwide survey of Japanese public opinion about off-label use of anticancer drugs recommended by comprehensive genomic profiling
Source: Int J Clin Oncol. 2025 Jul 18;30(9):1692–9. doi: 10.1007/s10147-025-02809-y (PMC12378634; doi:10.1007/s10147-025-02809-y)
Supplement: Supplementary file 2 — Supplementary file2 (PDF 77 KB) Supplementary Fig S2. Medical backgrounds and diseases of participants. A, The medical history of survey participants. Horizontal bars indicate the number of participants in groups of cancer patients (yellow), medical professionals (blue), and non-cancer volunteers (green). B, The distribution of cancer types in cancer patients. The numbers in the pie chart denote the number of patients. [file 10147_2025_2809_MOESM2_ESM.pdf]

Supplementary Fig. S2

A

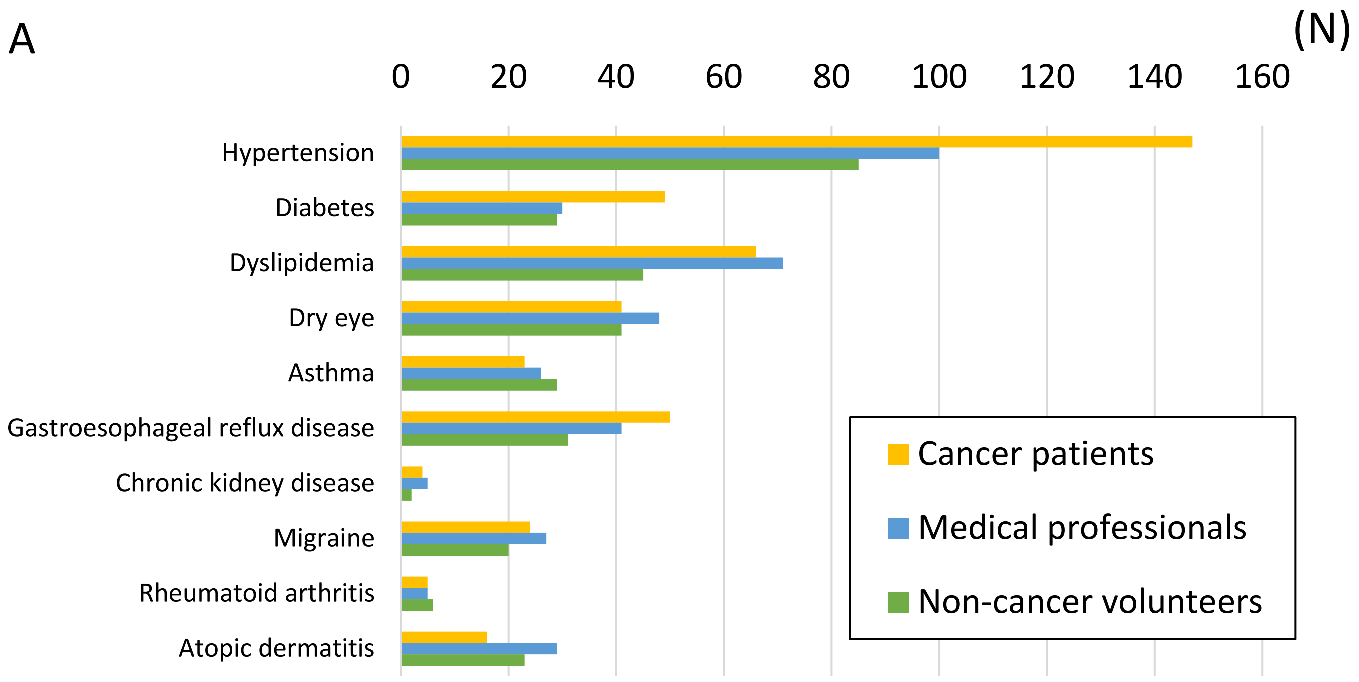

B

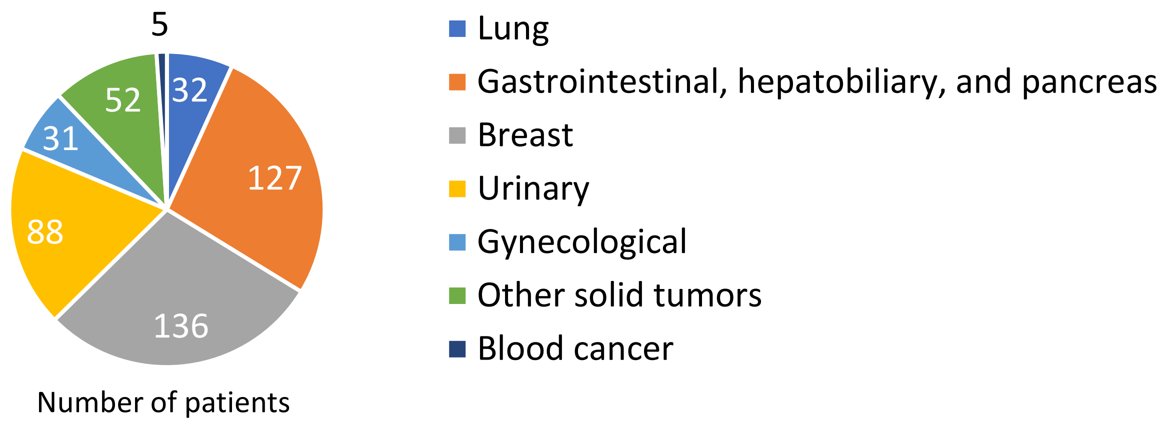

Supplementary Fig S2. Medical backgrounds and diseases of participants.
A, The medical history of survey participants. Horizontal bars indicate the number of participants in groups of cancer patients (yellow), medical professionals (blue), and non-cancer volunteers (green). B, The distribution of cancer types in cancer patients. The numbers in the pie chart denote the number of patients.
