# Supplementary material for: The nationwide survey of Japanese public opinion about off-label use of anticancer drugs recommended by comprehensive genomic profiling
Source: Int J Clin Oncol. 2025 Jul 18;30(9):1692–9. doi: 10.1007/s10147-025-02809-y (PMC12378634; doi:10.1007/s10147-025-02809-y)

Supplementary Figure S3

Supplementary Fig S3. Willingness to utilize off-label drugs according to overall comprehension of explanatory materials and cost burden.

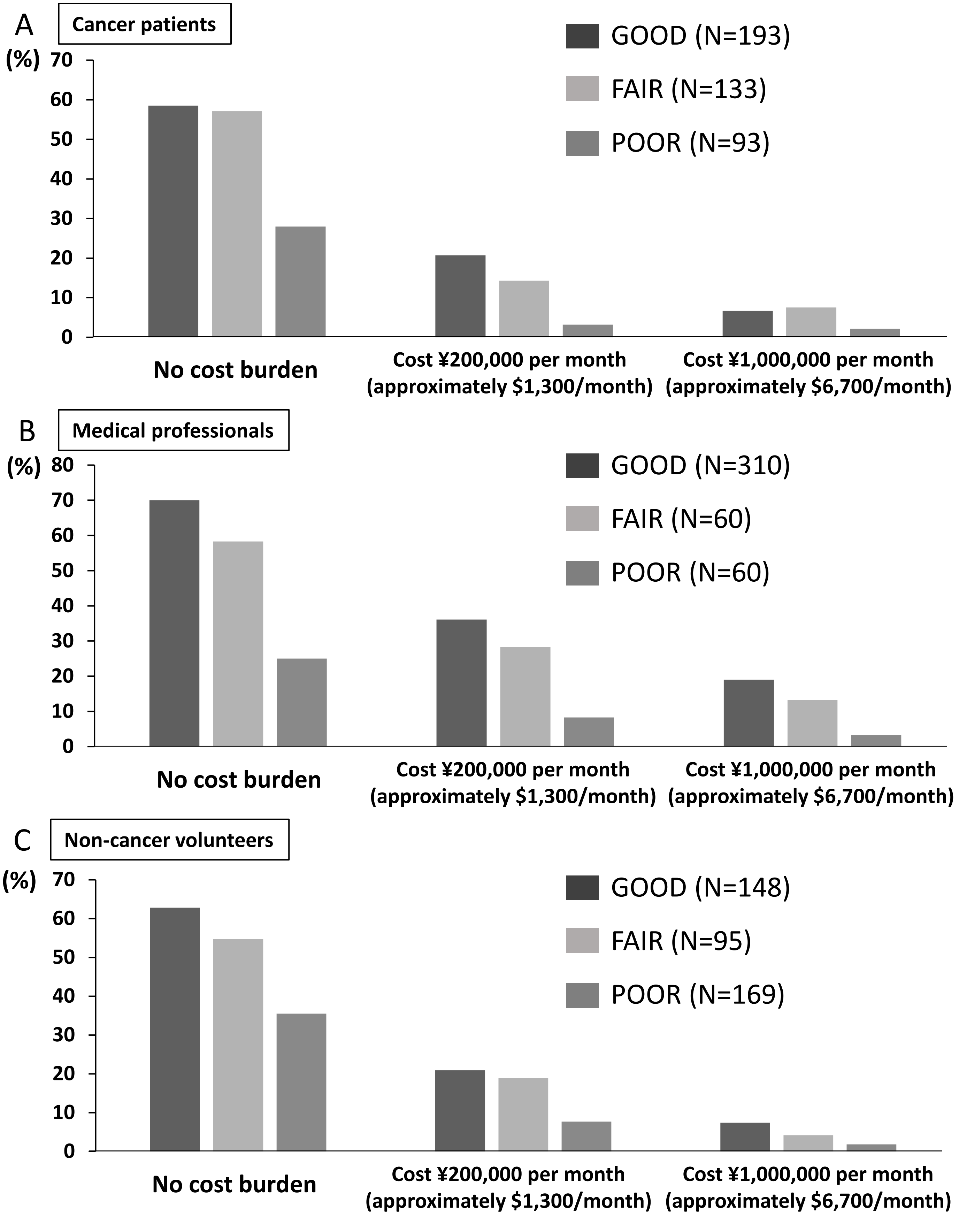

Supplement: Supplementary file 3 — Supplementary file3 (PDF 79 KB) Supplementary Fig S3. Willingness to utilize off-label drugs according to overall comprehension of explanatory materials and cost burden. Vertical bar graphs show the percentage of individuals willing to use the off-label drug in GOOD overall comprehension (dark gray), FAIR overall comprehension (light gray), and POOR overall comprehension (gray) according to the cost burden per month (zero, \200,000, and \1,000,000) in cancer patient participants (A), medical professional participants (B), and non-cancer volunteer participants (C). [file 10147_2025_2809_MOESM3_ESM.pdf]
